# PARO ROBOT INTERACTION DECREASES PAIN AND AGITATION SCORES IN HOSPITALIZED OLDER ADULTS WITH ADRD AND/OR DELIRIUM

**DOI:** 10.1093/geroni/igad104.2988

**Published:** 2023-12-21

**Authors:** Pamela Cacchione, Ellen Munsterman, Lisa Walke, Lisa Triantos, Michelle Johnson, Jodi Cheeks

**Affiliations:** University of Pennsylvania School of Nursing; Penn Presbyterian Medical Center, Philadelphia, Pennsylvania, United States; University of Pennsylvania, Philadelphia, Pennsylvania, United States; University of Pennsylvania, Philadelphia, Pennsylvania, United States; Penn Presbyterian Medical Center, Philadelphia, Pennsylvania, United States; University of Pennsylvania, Philadelphia, Pennsylvania, United States; Penn Presbyterian Medical Center, Philadelphia, Pennsylvania, United States

## Abstract

Hospitals are stressful for persons with Alzheimer’s Disease and Related Disorders (ADRD) and/or delirium. Therapeutic robotic animals, used in long-term care, are gaining traction in hospitals to decrease agitation. The purpose of this RCT was to 1) test the effectiveness of the PARO Robot in decreasing agitation (CMAI-OT) and pain, (PAINAD); 2) quantify the interactions (SIT) with PARO over 2 visits in persons with ADRD and/or delirium. We enrolled 45 intervention and 42 attention control (2 one-hour PARO interactions vs. research assistant interactions) participants. The CMAI-OT and PAINAD were completed at baseline and every 20 minutes X 3 in both groups. No differences between groups on age (M=83.7), gender (63% female), race (77 % Black) number of antipsychotics prescribed [M=1.2 vs M1.5, p=0.14], or baseline CMAI-OT scores PARO vs control [M=9.3 vs M=9.4, p=0.94]. CMAI-OT decreased at 20 minutes in the PARO group [M=3.9 vs. M=7.0, p= .004] and remained lower for the hour but not significantly. PAINAD scores were similar at baseline [M=2.0 vs M=2.2, p=0.65]. PAINAD scores decreased for PARO vs. control [M= 1.64 vs. M= 2.28, p=0.02; M=0.9 vs. M= 1.8, p=0.03] at 20 & 60 minutes day one without differences day two. Participants held PARO; engaged physically; talked to; smiled; and agreed to have PARO return. We demonstrated improved agitation for first 20 minutes of PARO interaction as well as lower pain over the first 60 minutes day one. Findings did not persist through day two. Next steps are to measure emotional responses to PARO.

